# Safety and rapid response of dabrafenib and trametinib therapy during hyperbilirubinemia in metastatic melanoma

**DOI:** 10.3389/fonc.2023.1102330

**Published:** 2023-02-14

**Authors:** Walid Shalata, Rachel Steckbeck, Ilya Polishchuk, Ahron Yehonatan Cohen, Keren Rouvinov, Margarita Tokar, Ashraf Abu Jama, Omar Abu Saleh, Kim Sheva, Alexander Yakobson

**Affiliations:** ^1^The Legacy Heritage Center & Dr. Larry Norton Institute, Soroka Medical Center and Ben Gurion University, Beer Sheva, Israel; ^2^Medical School for International Health, Ben Gurion University of the Negev, Beer Sheva, Israel; ^3^Internal Medicine Ward, Soroka Medical Center & Ben-Gurion University, Beer-Sheva, Israel; ^4^Department of Dermatology and Venereology, Emek Medical Centre, Afula, Israel

**Keywords:** dabrafenib, trametinib, hyperbilirubinemia, metastatic melanoma, BRAF mutation (V600E), safety

## Abstract

This case report describes the occurrence of hyperbilirubinemia as a complication of metastatic melanoma. A 72-year-old male patient was diagnosed with BRAF V600E-mutated melanoma with metastases in the liver, lymph nodes, lungs, pancreas, and stomach. Due to a lack of clinical data and specific guidelines for the treatment of mutated metastatic melanoma patients with hyperbilirubinemia, a conference of specialists debated between initiating treatment or providing supportive care. Ultimately, the patient was started on the combination therapy of dabrafenib and trametinib. This treatment resulted in a significant therapeutic response *via* normalization of bilirubin levels and an impressive radiological response of metastases just one month post-treatment initiation.

## 1 Introduction

The incidence of melanoma in high-risk populations is increasing around the world (1). Approximately 4% of all patients diagnosed with the disease each year suffer from stage 4 metastatic melanoma (2), The averaged 3-year overall survival (OS) rates were 41.3% for BRAF plus MEK inhibitor, 49.9% for PD-1 inhibition, and 58.4% for CTLA-4 plus PD-1 inhibition, in first-line therapy (3), a highly concerning statistic.

Melanoma is an extremely diverse disease with a variable pathogenesis that depends on body location as well as individual risk factors, such as UV exposure. This is attested by the fact that melanoma contains one of the highest tumor mutational burdens of all cancers, with an average of more than 10 mutations per megabase (4, 5). This is especially true in cutaneous melanomas arising from UV exposed areas (4). The majority of melanomas present with an upregulation of the MAPK/ERK signaling cascade (5), which is involved in cell proliferation, differentiation, and suppression of inflammation (6, 7).

The Cancer Genome Atlas Network has divided melanoma mutations into four sub-types being BRAF, RAS, NF-1, and triple-wild type (8). This categorization aids in the targeted treatment of melanomas. With the BRAF gene being the most commonly mutated gene in melanoma, the BRAF subtype has the highest occurrence rates (5). The vast majority of BRAF mutations have been identified in amino acid 600 of the gene, leading to the discovery of the V600E, V600K, and V600R point mutations (8, 9). Patients with BRAF subtype melanomas have been reported to be younger on average as compared to patient with other subtypes (8). However, BRAF mutations are usually not enough to induce tumors on their own, where most benign nevi also harbor BRAF mutations. The development of a melanoma requires an accumulation of various mutations, such as those in the PI3 kinase pathway, TERT, or CDKN2A (5, 10, 11)

Due to the relatively high frequency of BRAF mutations in melanoma, several targeted treatments have been developed. These treatments include dabrafenib and vemurafenib, which directly inhibit BRAF; and cobimetinib and trametinib, which inhibit MEK, the downstream kinase to BRAF and encorafenib and binimetinib (11). Given as a BRAF and MEK inhibitor combination these treatments generally have a very high response rate (up to 70%), giving patients an additional year of life on average (11). Meta-static melanoma may cause elevated bilirubin levels in patients, with the majority of re-ported cases being due to metastasis in the liver, pancreas, or common bile duct (12–17). Data on the exact changes in bilirubin levels upon treatment of metastatic melanoma, however are lacking. Herein, we present the first reported case, to the best of our knowledge, of bilirubin elevation in a patient with aggressive metastatic melanoma treated with combination therapy of dabrafenib and trametinib. The patient exhibited significant improvement in the widespread disease, highlighting the safety and efficacy of the treatment despite high levels of bilirubin. We believe that this case report may provide new insight for the management and treatment of metastatic melanoma.

## 2 Case report

A 72 year-old male was referred to the emergency department in May 2021 by a family physician due to hematemesis, jaundice, and a decreased performance status. The patient was an active smoker (30 packs per year for the last 40 years), but was generally healthy, was on no medications, and had no family history of cancer. Upon admission, the patient reported an 8 kg weight loss over the past month. A physical examination revealed jaundice of the skin, sclera, and mucous membranes, with no other pathological findings.

**Figure 1:**
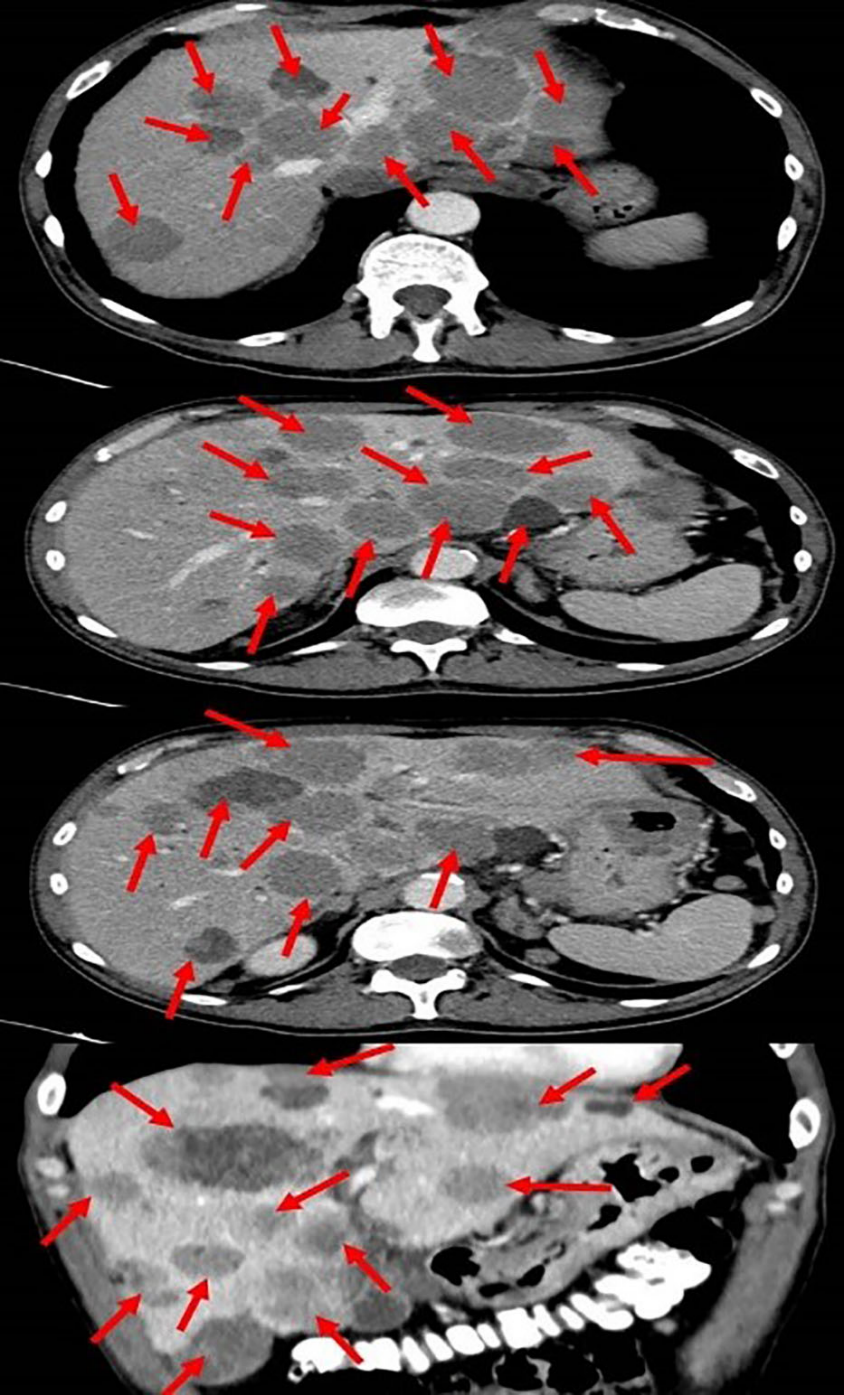
Total body computed tomography scan showing the metastatic melanoma in the liver (red arrows).

Initial blood tests revealed low hemoglobin levels of 7.1 gm/dL (normal range: 14-16 gm/dL), accompanied by mild leukocytosis and thrombocytosis. Blood chemistry showed an elevation in both total and direct bilirubin of 11.05 and 6.97 gm/dL respectively (nor-mal range: 0.3-1.2 gm/dL), and elevated liver enzymes, especially in alkaline phosphatase and gamma-glutamyl transferase (GGT). The lactate dehydrogenase (LDH) level was 1193 (IU/L). These results were consistent with obstructive jaundice, with the elevated levels of alkaline phosphatase and GGT suggesting intrahepatic cholestasis. Chest radiography showed small nodules in the left lung that were suspected of being metastatic processes. A total body computed tomography (CT) scan (**Figure 1**) revealed hepatomegaly (25*19 cm in diameter), several nodules in both lungs suspected of being metastasis (the biggest was 15 mm in diameter), enlargement of the lymph nodes in the hilum of both lungs, a space occupying lesion (SOL) in the vertebral area (D10), a SOL in the fundus of the stomach (6 cm in diameter), multiple SOLs in the liver (the biggest was 5 cm in diameter), enlargement of the lymph nodes in the hilum of the liver (the biggest 5 cm in diameter, SOLs in the head of the pancreas, SOLs in the bilateral adrenals, and several enlarged lymph nodes in the abdominal space.

**Figure 2:**
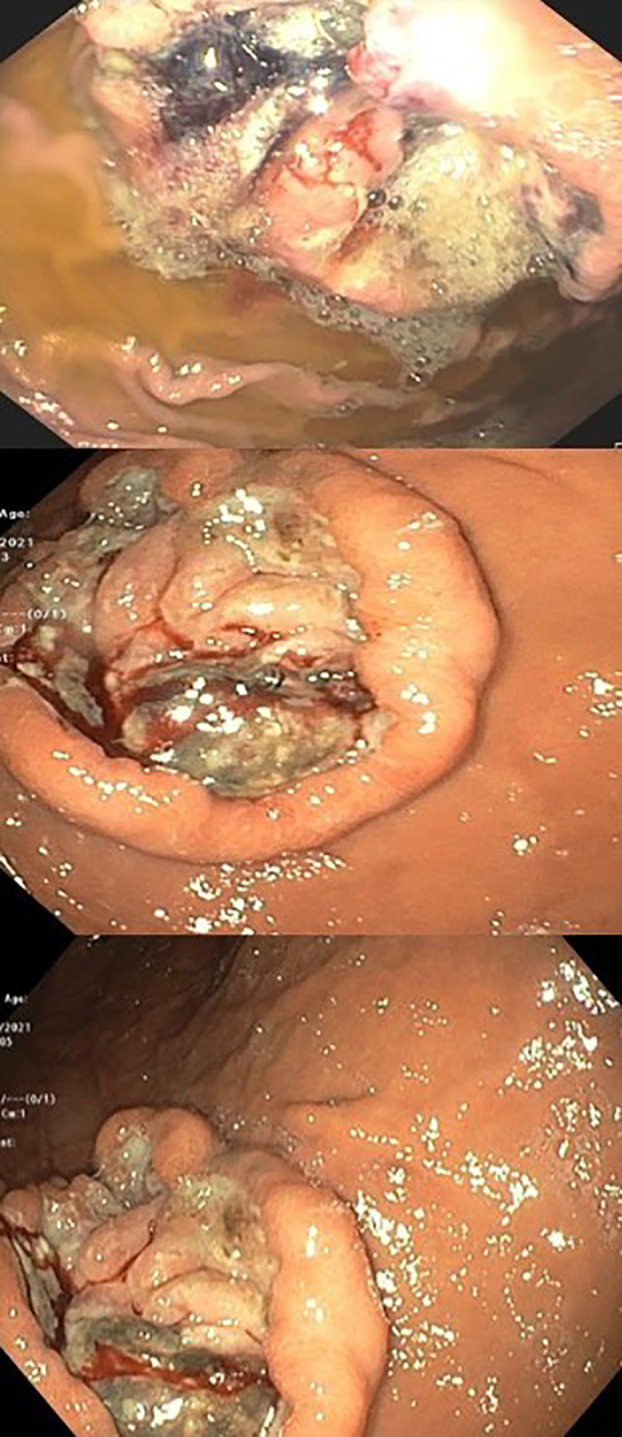
Pictures that were taken during gastroscopy which showing the active bleeding.

An ultrasound of the upper abdomen was performed and a common bile duct obstruction or dilatation were ruled out. The patient was sent for an urgent gastroscopy, which showed four peptic ulcers in the stomach (2 ulcers in the antrum, 1 ulcer in the body and 1 ulcer in the fundus) (**Figure 2**).

**Figure 3:**
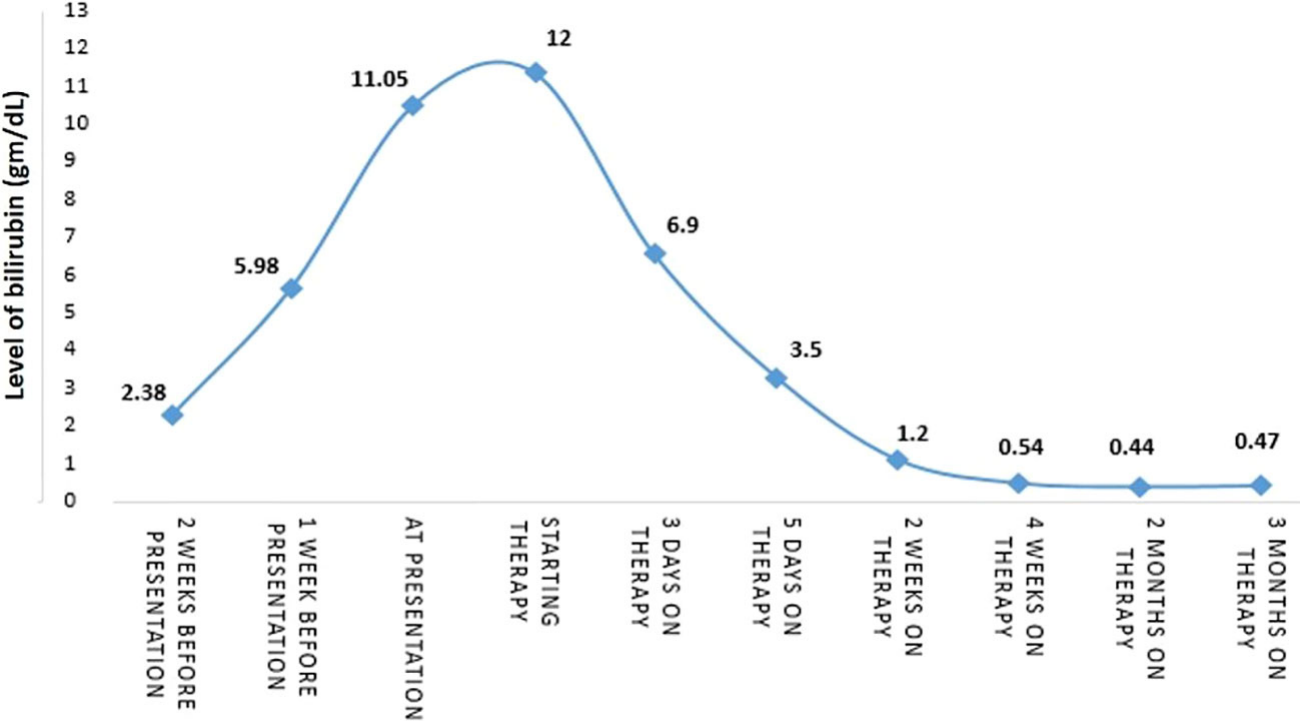
The patient’s timeline for cancer treatment and bilirubin levels.

The biggest ulcer was 6 cm in diameter and was actively bleeding. A biopsy was taken from the ulcers, and histopathological results showed malignant melanoma. Molecular testing revealed the BRAF V600E mutation. The presumptive clinical diagnosis was stage 4 metastatic melanoma and a multidisciplinary conference was held which included an oncologist, hepatologist, and radiologist who all recommended systemic therapy for this extremely aggressive disease, despite very high bilirubin levels (had increased to 12 gm/dL). The possibility of stent insertion was discussed and rejected by the forum after careful assessment of imaging tests. The patient started combination therapy for his mutation (BRAF) with dabrafenib (150mg twice a day daily) and trametinib (2mg once a day daily). Three days after treatment initiation, bilirubin levels decreased to 6.9 gm/dL. Two days later levels decreased to 3.5 gm/dL, and one week later (2 weeks since treatment initiation), levels decreased to just above the normal range (1.2 gm/dL). After two weeks (al-most a month after starting treatment), bilirubin levels were within the normal range (0.54 gm/dL) (**Figure 3**).

**Figure 4:**
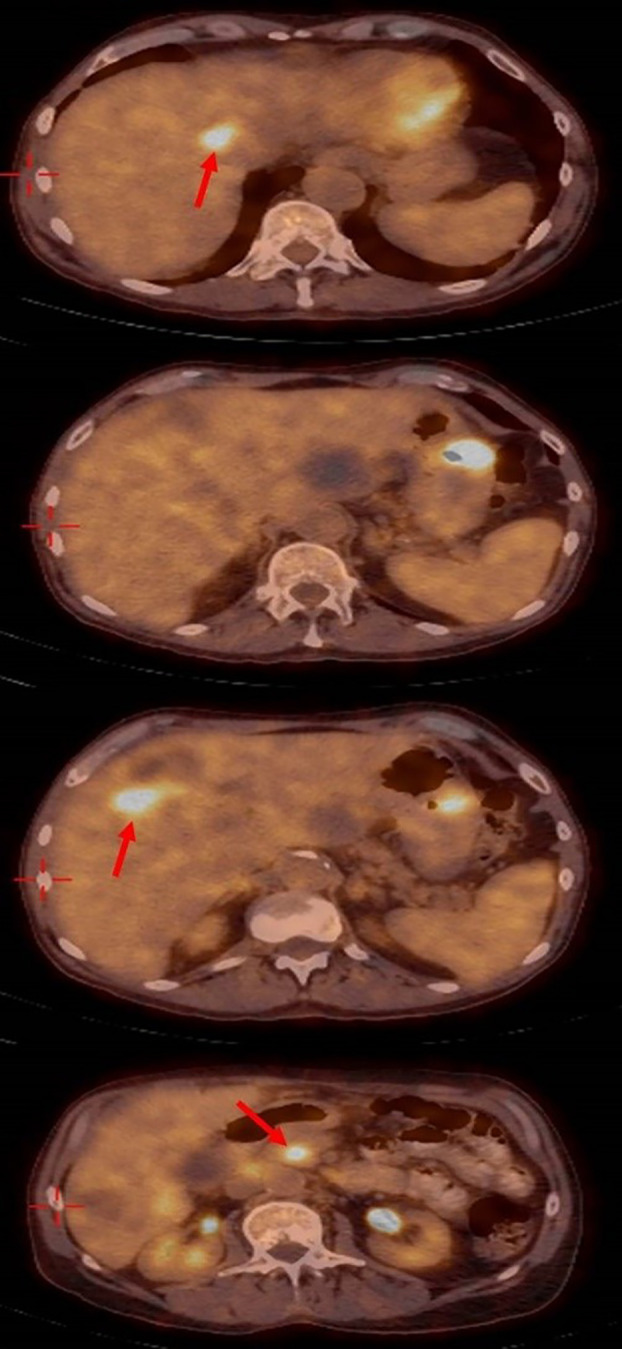
Positron emission tomography– computed tomography showing the significant response with disappearing of most liver metastasis.

One month later a positron emission tomography PET– CT scan (**Figure 4**), showed a significant improvement with the disappearance of the lung nodules, lymph nodes in the hilum of both lungs being of normal size, the disappearance of the SOL in the D10 area, decreased diameters of the SOLs in the head of the pancreas, a decreased diameter of the SOL in the fundus of the stomach (to 2.8cm), the disappearance of some of the SOLs in the liver, and decreased diameters of the lymph nodes in the liver hilum. No SOLs or enlargement in bilateral adrenals or lymph nodes of the abdominal space were seen.

The patient remained on follow-up status with routine blood tests, which remained within normal range 6 months from treatment initiation. Unfortunately he died due to disease progression in February 2022 without additional follow-ups.

## 3 Discussion

This case report describes a patient with serious complications related to metastatic melanoma. The patient suffered from widespread metastatic disease, accompanied by very high levels of bilirubin due to intrahepatic cholestasis caused by liver metastases. Elevated bilirubin levels are due to one of three main causes: 1. Pre-hepatic complications such as a massive breakdown of red blood cells (hemolysis); 2. Hepatic causes, such as direct damage of the liver cells (toxins, viral infections and metastatic disease); and 3. Post-hepatic causes such as gallstones and locally advanced cancers (18). In addition, the patient suffered from elevated LDH levels, seven organ sites with metastases, and a very poor ECOG score of 4. Without immediate intervention, it was expected that this patient would not survive. Expected treatment outcomes remained unknown.

According to the ASCO guidelines on systemic treatment of melanoma, patients with BRAF-mutant melanoma stage III should be treated with either nivolumab, pembrolizumab or a combination dabrafenib and trametinib therapy (19). For patients with stage IV unresectable or metastatic melanoma, more options are available, including immunotherapy (ipilimumab plus nivolumab, nivolumab or pembrolizumab) or targeted therapy for BRAF-positive patients (dabrafenib plus trametinib, encorafenib plus binimetinib or vemurafenib plus cobimetinib) (19).

Dabrafenib is an inhibitor of the associated enzyme BRAF. Primary routes of elimination of dabrafenib are hepatic metabolism and biliary secretion. Whereas mild hepatic impairment is not a contraindication for administration of dabrafenib, there is no available data regarding the safety and efficacy of this therapy among patients with severe hepatic impairment.

Trametinib is a MEK inhibitor drug with anti-cancer activity. It inhibits MEK1 and MEK2. According to the manufacturer, moderate or severe hepatic impairment had no significant effect on trametinib exposure or apparent clearance.

The combination of dabrafenib and trametinib was approved for use in BRAF V600-mutant melanoma in 2013 (20). It is known to have a fast response with improved survival rates among patients compared to that of BRAF inhibitor monotherapy (21). However, during trials, these treatments did not include patients with high levels of bilirubin, and thus patient response in such an instance is relatively unknown. This combination however has been shown to be more efficacious in the treatment of melanoma than dabrafenib alone (22). During clinical trials, several side effects were reported, including pyrexia, chills, fatigue, nausea, vomiting, diarrhea, headache, rash, arthralgia, and myalgia (20, 23).

Due to the aggressive nature of metastatic melanoma disease and this specific patient’s poor performance status, with a terminal prognosis without intervention. It was therefore decided that the best treatment option in this case would be combination target therapy with dabrafenib and trametinib. This treatment option was very effective, and the patient showed rapid improvements in bilirubin levels and in radiographic findings of metastases.

One initial issue with BRAF-mutation-specific treatment is that resistance to single drug regimens may develop quickly, usually within 6-7 months of beginning treatment (9, 24). Therefore, regimens consisting of two drugs, i.e., a BRAF inhibitor and a MEK inhibitor, have been recommended and approved. Although such combinations do not eliminate the progression of resistance, they do delay it (24).

Jason et al. showed that patients with BRAF mutant melanoma with liver metastases treated with targeted therapy, had higher proportion of response in the liver metastases (46.3%) compared with patients treated with immunotherapy (35.0%) (25).

Recently several clinical trials evaluated patients with unresectable or metastatic BRAF-mutant melanoma with the combination of BRAF/MEK inhibitors with anti-PD-(L)1 therapy known as triplet therapy (KEYNOTE-022, IMspire150 and COMBI-i). These trials showed that the objective response rate of KEYNOTE-022 was surprisingly lower (63%) compared to the control arm (72%), and unsatisfactory results were seen in the IMspire150 (66.5% for triplet therapy compared to the control arm 65%). COMBI-i showed better results but not convincingly (68.5% for triplet therapy compared to the control arm 64.2%). However, all three trials showed a trend of improved overall survival (26–29). Furthermore, all three trials showed a significant increase in toxicity that were reported in the triplet arms compared with the combination arms, especially with higher rates of treatment-related toxicity- grades 3–5, such as treatment-related death, elevated creatine phosphokinase, rate of treatment discontinuation, hepatitis, pneumonitis, fever, rash, diarrhea and liver transaminase elevation (27–29).

## 4 Conclusions

In summary, we report a case of a patient with complicated hyperbilirubinemia, anemia and hypertransaminasemia related to metastatic melanoma, the patient had BRAF mutant (V600E), because of his aggressive hyperbilirubinemia and performance status it was very hard to choose. However, for a lifesaving situation we decided an important strategy for treatment decision-making, therefore, the patient was treated with BRAF and MEK inhibitor combination (dabrafenib and trametinib). The patient achieved significantly radiological and blood test response. Overall, our case demonstrates the importance and potential of the safety for treating hyperbilirubinemia and hypertransaminasemia in patients with metastatic melanoma with BRAF mutant.

## Data availability statement

The original contributions presented in the study are included in the article/supplementary material. Further inquiries can be directed to the corresponding author.

## Ethics statement

Ethical review and approval was not required for the study on human participants in accordance with the local legislation and institutional requirements. The patients/participants provided their written informed consent to participate in this study.

## Author contributions

Conceptualization, WS and AY; methodology, WS; software, WS; validation, WS, AY, RS, AC, KR, MT, AO, AA, IP, and KS; formal analysis, WS; investigation, WS, AY, RS, AC, KR, MT, IP, AO, AA, and KS; resources, WS; data curation, WS; writing—original draft preparation, WS, and RS,; writing—review and editing, WS, AY, RS, AC, KR, MT, AO, AA, and KS; visualization, WS; supervision, WS; project administration, WS; funding acquisition, WS. All authors contributed to the article and approved the submitted version.
